# Health-related quality of life in cancer patients at the end of life, translation, validation, and longitudinal analysis of specific tools: study protocol for a randomized controlled trial

**DOI:** 10.1186/1745-6215-13-39

**Published:** 2012-04-20

**Authors:** Anne-Lise Poirier, Fabrice Kwiatkowski, Jean-Marie Commer, Bénédicte D'Aillières, Virginie Berger, Mariette Mercier, Franck Bonnetain

**Affiliations:** 1 Centre d'Evaluation Clinique en Oncologie, Centre Paul Papin, Angers, France; 2 Unité de biostatistiques, Centre Jean-Perrin, Clermont Ferrand, France; 3 Laboratoire de biostatistiques, CHU Jean Minjoz, Besançon, France; 4 Unité de soins palliatifs et de soins de support Centre Paul Papin Angers, Angers, France; 5 Unité de biostatistiques et d'épidémiologie, EA 4184. Centre Georges François Leclerc Dijon, Dijon, France; 6 Plateforme de recherche clinique Qualité de vie et cancer, Dijon, France

**Keywords:** Quality of life, End of life, Cancer

## Abstract

**Background:**

The end of life for cancer patients is the ultimate stage of the disease, and care in this setting is important as it can improve the wellbeing not only of patients, but also the patients' family and close friends. As it is a matter of profoundly personal concerns, patients' perception of this phase of the disease is difficult to assess and has thus been insufficiently studied. Nonetheless, caregivers are required to provide specific care to help patients and to treat them in order to improve their wellbeing during this period.

While tools to assess health-related quality of life (QoL) in cancer patients at the end of life exist in English, to our knowledge, no validated tools are available in French.

**Methods/design:**

This randomized multicenter cohort study will be carried out to cross-culturally adapt and validate a French version of the English QUAL-E and the Missoula Vitas Quality Of Life Index (MVQOLI) questionnaires for advanced cancer patients in a palliative setting. A randomized clinical trial component in addition to a cohort study is implemented in order to test psychometric hypotheses: order effect and improvement of sensibility to change.

The validation procedure will ensure that the psychometric properties are maintained.

The main criterion to assess the reliability of the questionnaires will be reproducibility (test-retest method) using intraclass correlation coefficients. It will be necessary to include 372 patients. The sensitivity to change, discriminant capability as well as convergent validity will be also investigated.

**Discussion:**

If the cross-cultural validation of the MVQOLI and QUAL-E questionnaires for advanced cancer patients in a palliative setting have satisfactory psychometric properties, it will allow us to assess the specific dimensions of QoL at the end of life.

**Trial registration:**

Current Controlled Trials NCT01545921.

## Background

The end of life for cancer patients is the ultimate stage of the disease. However, in this setting, improving quality of life (QoL) and the wellbeing of patients should remain a major concern. Given the profoundly personal concerns involved, patients' perceptions of the end of life are difficult to assess and have therefore been insufficiently studied. Clinicians, nurses, and other paramedical personnel are required to provide individualized care adapted to the end of life in order to improve patients' QoL and wellbeing [1,2].

Today, there is no consensus to define the time frame related to the 'end of life' of a cancer patient [3]. For example, depending on the cancer site, the 'palliative' period could vary from a few days to a few months. Moreover, the palliative and 'end-of-life' periods could overlap, but the therapeutic goals for these two periods should not be the same. In order to provide optimal care, it seems crucial to distinguish between these two periods as the former has a more therapeutic goal while the latter is to accompany patients as they near death. This ultimate stage of disease could require caregivers to meet new demands not only from patients, but also from their relatives.

To our knowledge (Patient Reported Outcome and Quality Of Life Instruments Database) [4-6] there are no French validated tools to assess health-related QoL for cancer patients at the end of life. QoL is a major endpoint to assess therapeutic strategies in cancer patients, particularly in a palliative setting when no benefits in overall survival can be expected. In this context, QoL should be considered the primary endpoint for trials [7,8].

QoL at the end of life could be evaluated using generic QoL questionnaires like EORTC QLQ-C30 and/or the Medical Outcome study Short Form 36 (SF-36) or by using questionnaires focusing on symptoms. For example, the Edmonton Symptoms Assessment System (ESAS) is a dedicated tool to assess pain [9]. Other tools allow caregivers to assess depression [10] or fatigue [11].

Some studies have highlighted the fact that additional specific items could be required to take into account the most important QoL dimensions or symptoms at the end of life such as symptom and pain management [12-16], quality of care [15,16], preparation for death [12,13], relationships/communication [12-14], family context [16], dignity [12,16], choice and control [12] the financial aspect of care [17,18], completion [18], or spirituality/transcendence [14-16,18].

Current tools to assess QoL do not explore these specific dimensions, which could capture major aspects of QoL at the end of life.

Some questionnaires dedicated to the palliative setting have been proposed, but they are too long, and\or specific domains related to the end of life are not explored. Validated QoL tools in French are even more restricted; only the Mc Gill [19,20] Support Team Assessment Schedule (STAS) [21] and QLQ-C15 PAL questionnaires (shortened version of QLQ-C30) [22] are available. Moreover, these tools are more specifically dedicated to the palliative setting.

The Missoula-Vitas Quality Of Life Index developed by Byock *et al*. has been specifically designed for the end of palliative setting [14]. This QoL tool provides an exhaustive assessment of major dimensions in that setting. The shortened version included only 15 items investigating five dimensions: symptoms, function, interpersonal relationships, wellbeing, and transcendence [14]. Another tool, the QUAL-E, is longer and includes 25 items that investigate five domains: life completion, relations with the healthcare system, preparation for end of life, symptom severity, and affective social support [18]. However, these QoL tools have not been adapted to French culture and translated into French. Another concern is that they are not specific to cancer patients.

The first step to improve evaluations of how French patients perceive this disease setting is to translate the available specific 'end-of-life' QoL tools. Assessing QoL at the end of life with dedicated tools could help to compare therapeutic strategies and could improve care in the palliative setting. Based on these hypotheses, we propose to adapt QoL tools dedicated to the end of life to French culture and to validate them for French cancer patients.

## Methods

### Objectives

Primary objectives are: to cross-culturally adapt the English questionnaires QUAL-E and MVQOLI (Missoula Vitas Quality Of Life Index) and to validate their psychometric properties in the end-of-life setting.

Intermediate objectives are: to translate and adapt the English version of QUAL-E and MVQOLI into French taking into account cultural differences.

Secondary objectives are: to longitudinally assess QoL of cancer patients until death using the QLQ-C15 PAL, the French version of QUAL-E and the Missoula Vitas Quality of Life Index (MVQOLI); to assess the impact of therapeutic strategies on QoL at the end of life; to assess the added value of spontaneous completion of QoL questionnaires to detect a clinically significant change in this setting; to assess the effect of the order of questionnaires on QoL levels; to define a time period for the 'end of life'.

Primary outcomes for cross-cultural and psychometric validation (see also statistical methods): reproducibility of QoL scores estimated by intraclass correlation coefficients; sensitivity to change estimated by effect size and the standard error of mean; discriminant capability; and convergent validity.

### Study design

We propose to perform a prospective multicenter cohort study. A randomized clinical trial component in addition to a cohort study is implemented in order to test psychometric hypotheses related to order effect and to the improvement of sensibility to change. This study has been designed in accordance with the validation methods of the FDA and the International Society For Pharmacoeconomics and Outcomes. It was reviewed by a national patients' committee in July 2009. The Clinical trial was registered to the French Safety Agency for Health Products (AFSSAPS, that is the Health Authority in France). Although this study does not assess health products, it was registered as biomedical research. The authorization was in agreement with the Article L. 1123-8 Code of Public Health and was obtained in February 2010.

This clinical trial was registered at AFSSAPS (2010-A00196-33). The project was approved by the local ethics committee (CPP Ouest II Angers) in April 2010.

The Study was registered in March 2012 on ClinicalTrials.gov; the registration number is NCT01545921.

The project received a grant so-called PHRC (Programme Hospitalier de Recherche Clinique 2011) in 2011 from the French national cancer institute (INCa, Institut National du Cancer).

### Population

To be eligible, patients should have been diagnosed with advanced cancer (with any cancer site) and treated or not with palliative intent (chemotherapy, analgesic radiotherapy, surgery without curative intent), with an ECOG or a WHO performance status ≥ 2, a life expectancy ≥ 1 month, and an age ≥ 18 years.

Patients should have been informed about the palliative stage of the disease and should have been followed for at least 1 month by a palliative caregiver to be included in the study.

The non-eligibility criteria are: a psychiatric disease that compromises understanding of the study objectives and/or informed consent and/or the inability of patients to follow the study design for psychological, social, family, or geographical reasons.

Written informed consent is required before inclusion.

### Recruitment

Once eligibility criteria have been checked, patients from French comprehensive cancer care centers or hospitals will be invited to join the study.

Before inclusion, patients should read and understand the study information letter and then should date and sign the informed consent form (ICF) to allow them to join the study.

The coordinating center will be notified of each inclusion before completion of the first QoL questionnaire (inclusion document will be completed and returned to Paul Papin Center, Angers by fax).

Since eligibility criteria will be checked and patients' ICF will be collected, the first QoL questionnaires and the test-retest (3 days after the first completion) will be completed (before randomization) in the following order: QLQ-C15 PAL, MVQOLI, and QUAL-E.

After completion of the retest QoL questionnaire, the eligibility criteria and date of signed informed consent will be collected on a dedicated internet site to allow e-randomization with a personal log-in and password (Tenalea software). The study coordinator will then be able to randomize patients. The allocated arm will be sent to investigators, to the methodologists, the principal investigator and the coordinating center by email.

Patients will be randomized after the test-retest to limit the number of non-assessable patients for reproducibility.

### Randomization

To explore secondary objectives 3 and 4 using a factorial design, QoL questionnaire order, and spontaneous QoL completion will be randomized 1:1:1:1 using a minimization technique stratified according to center, cancer location, sex, and age.

Patients will be randomized in one of the following four arms (Table 1): (1) Arm A: MVQOLI then QLQ-C15 PAUL and QUAL-E, evaluation every month; (2) Arm B: QLQ-C15 PAL, MVQOLI and QUAL-E, evaluation every month; (3) Arm C: MVQOLI then QLQ-C15 PAL and QUAL-E, evaluation every month and spontaneous QoL completion; and (4) Arm D: QLQ-C15 PAL, MVQOLI and QUAL-E evaluation every month and spontaneous QoL completion.

**Table 1:** Follow-up for QoL and data collection according to randomized arms

	Baseline		Follow-up	
	**D1^a^**	**Between** **D1 and D3^b^**	**Before every consultation until death (J30+/-3 days)**	**If necessary according to randomized allocated arm (notebook)**

**Collected data and order of administration for QoL questionnaires**				

	**Arms**	**Arms**	**Arms**	**Arms**

Consent	A/B/C/D			

Questionnaires: QLQ-C15, MVQOLI and QUAL-E	A/B/C/D	A/B/C/D	B and D	

Questionnaires: MVQOLI, QLQ-C15 and QUAL-E			A and C	

Questionnaires: MVQOLI, QLQ-C15				D and C

Performance Status, Performance Palliative Scale	A/B/C/D	A/B/C/D	A, B, C, D	

Vital signs, pain (EVA)	A/B/C/D	A/B/C/D	A, B, C, D	

Care (Oncology, Psychology, and so on)	A/B/C/D	A/B/C/D	A, B, C, D	

Responsiveness question	A/B/C/D	A/B/C/D		D and C

^a^D1: First day, the day where the first QoL questionnaires will be completed; D3: 3 days after the first day administration

^b^Can be done D1 at the end of the day if the first was done early in the day

^c^Only MVQOLI and QUAL-E

The above randomization was chosen to explore methodological aspects to optimize QOL tools in the end-of-life setting.

It will allow us to assess the occurrence of an order effect on QoL level by investigating a halo effect and/or a framing effect. Since every patient should complete three questionnaires (MVQOLI, QUAL-E, and the QLQ-C15 PAL), completion of the first questionnaire could influence responses for the following questionnaires, resulting in different QoL levels. Moreover, this effect could be differential depending on the completion order. Since QUAL-E needs a face to face interview, it will be completed by the patient at the end for each arm.

The second randomization will investigate the added value of spontaneous QoL completion to capture clinically important changes in QoL that could have occurred between two planned QoL assessments.

For each allocated group a specific case report form (CRF) will be produced with the corresponding order of QoL questionnaires to prevent changes in completion order (that is, 'cross-over') after the randomization.

Furthermore, the date and time of completion for each QoL questionnaire will be collected. This will allow us to check the order of completion used by the patients.

### Evaluation criteria

#### End of life QoL questionnaires

Within the framework of this study, three questionnaires will be used: QLQ-C15 PAL, QUAL-E, and MVQOLI. The McGill questionnaire was not retained since it assesses the QoL during a short period (quality of life during the 2 days before questionnaire completion).

The EORTC QLQ-C15 PAL reflects patients' QoL during the previous week and investigates two domains (symptoms and physical functioning). An additional item assesses patients' perception of overall QoL. This is a shortened version of the EORTC QLQ-C30, which aims to prevent missing QoL data due to patients' health status. Scores will be calculated using the same algorithm as that used in the QLQ-C30 methodology [22]. Scores will therefore range from 0 (poor QoL) to 100 (best QoL). [23,24].

Taking into account the limited number of dimensions investigated by the EORTC QLQ-C15 PAL, we have selected the QUAL-E and MVQOLI to complement QoL evaluations at the end of life. These two questionnaires dedicated to the end of life have been methodologically validated in their original language, English [25,26].

Twenty-five items and one overall question constitute the QUAL-E questionnaire. Five dimensions can be explored: life completion (particularly through contributions to others), relations with the healthcare system (knowledge and participation of patients in therapeutic decisions), preparation for the end of life, symptom severity (by capturing symptom impact), and affective social support. This questionnaire should be completed during a face-to-face interview. Every question is scored on a 5-point Likert scale [25,26].

The MVQOLI, developed by Byock *et al*. in 1995 [14,26], includes 15 items in the shortened version and 25 items in the original version. The shortened version was selected since Byock *et al*. previously showed a good correlation (r = 0.93) between these two versions, suggesting that the shortened version does not seem to be associated with a loss of information [14]. This tool explores five dimensions: symptoms (experience of the physical discomfort associated with progressive disease), functional activities (perceived ability to perform usual functions and activities), interpersonal (degree of investment in personal relationships and the perceived quality of one's relations with family and friends), wellbeing (contentment or lack of contentment with self), and transcendence (degree of experienced meaning and purpose in life). It helps professionals to identify factors that affect patient QoL at the end of life. The items related to the 'evaluation' range from -2 to +2, those for the 'satisfaction' range from -4 to +4 and those for 'importance' range from 1 to 5. Each dimension of the questionnaire will be calculated using specific items related to'satisfaction', 'evaluation', and 'importance'.

The score is the sum of the 'evaluation' and 'satisfaction' scores multiplied by the 'importance' score. The total score is obtained by summing each score related to each dimension divided by 10 and then adding 15 points. If the score is negative QoL is poor. The score reflects the impact of each dimension for patients.

### Completion of QoL questionnaire

The QoL questionnaires will be completed before randomization and then every month until death in a time driven design fashion.

Using test-retest methodology, the QLQ-C15 PAL, MVQOLI, and QUAL-E questionnaires will be completed 3 days after the first completion at baseline and before randomization [27].

Though a maximum delay of 15 days is usually allowed between completions to check reproducibility, since these patients have a specific end-of-life profile with sometimes a rapid deterioration in general health status, a maximum delay of 3 days was chosen to minimize the 'risk' of changes in health status.

Since the QUAL-E questionnaire should be completed using a direct interview; patients should complete it last to prevent social desirability bias on QLQ-C15 PAL and MVQOLI evaluations [27].

In order to prevent missing QoL data [28], if a patient is unable to complete questionnaires due to poor health status, QLQ-C15 PAL and MVQOLI could be completed with the help of a proxy (from the medical team) who will have to record the patient's answers without interpretation [29]. A specific item will be completed to take this information into account for the longitudinal analysis of QoL.

All patients will complete QoL questionnaires every month until death.

In arms C and D, depending on patients' health status or perceived QoL, patients will be free to complete the QLQ-C15 PAL and MVQOLI questionnaires (spontaneous QoL completion in an event drive design) between two planned evaluations. A patient CRF including QLQ-C15 PAL and MVQOLI questionnaires, the date of completion, the perception of QoL changes using the Jaescke transition question [30]), and a space for free comments will be created. This booklet will be given to the patients after randomization.

The QoL questionnaires will be administered in a homogeneous way by the same person, a nurse specialized in palliative care, a psychologist, or if necessary a clinical research assistant (CRA), throughout the patient follow-up until death.

Completed questionnaires should be returned to one of the CRAs assigned to the study. In order to deal with holidays, a minimum of two full-time CRAs will be required and identified for each participating center (Table 2).

**Table 2:** Center's participation agreement

Organization	City
Cancer care center Paul Papin	Angers, France

Cancer care center Léon Bérard	Lyon, France

Cancer care center Georges François Leclerc	Dijon, France

University hospital center Jean Minjoz	Besançon, France

Cancer care center Henri Becquerel	Rouen, France

Hospital center	Cholet, France

Cancer care center Val d'Aurelle-Paul	Montpellier, France

Cancer care institute Bergonié	Bordeaux, France

Cancer care center Oscar Lambret	Lille, France

Cancer care center François Baclesse	Caen, France

Cancer care institute curie	Paris, France

University hospital center	Angers, France

Original CRF and QoL questionnaires will be kept by the Paul Papin Center; while a copy will be kept on site. Postal delivery could be allowed.

If a patient does not want to or cannot complete QoL questionnaires, a note will be made on the unfilled questionnaire with a brief explanation. An item to collect reasons for non-completion should be also completed by CRAs.

Other data will be collected: demographic data, medical history, medical follow-up (social, psychological, pain, cancer treatment), clinical status, and Jaeschke transition question to define minimal clinically important difference for patients in this setting [30].

### Methodology

#### Translation of the English questionnaire

First step

The QUAL-E and MVQOLI were translated between May 2010 and July 2010 by the national '*Qualité de vie et cancer*' clinical research platform which had selected national experts to perform this initial step. A translation group (eight persons) including methodologists, linguists, psychologists, and practitioners in palliative care was created.

The translation into French and validation of the English-language QUAL-E and MVQOLI questionnaires were not done using backwards translations. Some studies underlined that backwards translation methods could generate a 'word by word' translation instead of fluent, natural, and appropriate language [31].

The selected translation process for linguistic validation included a translation to the target language (French) made by one translator who was a native English speaker. This allowed us to take into account specific intercultural validity problems.

Second step

Before initiating the prospective study, a pre-test will be done in 30 patients to check this translation. Particular attention will be paid to collecting data on patients' understanding of the questions as well as their acceptability. Patients' suggestions and their feelings regarding the QoL questionnaires will be collected.

Furthermore, clinicians specialized in palliative cancer care will also read and comment the proposed French translation.

This step is crucial to assess the cultural adaptation and the weight of the words in this setting. This step is actually ongoing. Agreements with the investigating center have been drafted (November 2010).

### Sample size

#### Primary endpoint

To study the validity of the questionnaires, the selected primary outcome is reproducibility assessed by intraclass correlation coefficients (ICC).

Taking into account the number of dimensions for each QoL questionnaire as well as the number of scores (continuous variable) for which the ICC will be calculated, using an unilateral alpha type one error of 0.001, a power of 80% and the following hypotheses (N'Query:Advisor V7):

H0: an ICC < 0.5 is uninteresting

H1: an ICC ≥ 0.65 is the minimum required for reproducibility

It will be necessary to include 338 patients.

Taking into account that 10% of patients will be lost to follow-up, it will be necessary to include 372 patients.

### Sample size for secondary objectives

The main endpoint to assess the added value of spontaneous QoL completion is the proportion of patients who report at least one Minimal clinically important difference (MCID) using the Jaeschke transition question.

The minimal clinically important difference is defined as an improvement or a deterioration of 5% in the theoretical QoL score range [23].

With a bilateral alpha type one error of 1% (Bonferroni adjustment for multiple comparisons) a power of 90%, and the following hypotheses:

H0: 70% of patients report an MCID before death in arms A and B

H1: 90% of patients report an MCID before death in arms C and D

It will be necessary to have 243 patients with at least two completed QoL questionnaires between two follow-ups representing 65.4% of the 372 patients included for the primary objective.

### Statistical analyses

A statistical analysis plan will be written before the database is locked.

The population and clinical characteristics will be described (sex, age, family situation, cancer site).

The rate of QoL completion will be described for each questionnaire at each follow-up. QoL scores will be described at each follow-up.

Continuous variables will be described by means (standard deviation) and median (minimum-maximum). Qualitative variables will be described by frequencies and percentages.

#### Primary objective analyses

This study will respect the FDA psychometric validation methods [7].

The first objective is to validate translation of QUAL-E and MVQOLI by checking the psychometric properties.

1. Validity [32-34]: Face validity and content validity: The translated questionnaires will be analyzed by patients and experts to check that the items of the questionnaires are in line with to the original version (major domains treated, question ambiguity). This step was conducted by the '*Qualité de vie et cancer' *platform experts group during cross-cultural translation of the English questionnaires.

Criterion validity: We will check that the translated tools correlate with reference QoL or clinical criteria by calculating correlation coefficients. QLQ-C15 PAL will be treated as the gold standard for QoL tools. A strong correlation between the measures given by the questionnaires and those given by the QLQ-C15 PAL will constitute proof of criterion validity. We will also use the WHO performance status and the performance palliative scale (PPS). These variables will be also dichotomized to calculate the sensitivity and specificity of QoL tools using ROC curves.

Construct validity: This psychometric property will be checked by calculating establishing convergences and differences with the other variables. Factorial analyses as well as Item Response theory methods will be used.

2. Reliability [32-34]: Effect size and the standardized response mean will be computed between each follow-up and then analyzed according to Cohen's classification.

Sensitivity to change:

Internal consistency: we will estimate the strength of the inter-correlations between items, and validate underlying dimensions. We will calculate Cronbach's alpha as an estimator of this internal consistency for each dimension of the QoL questionnaires (at each time assessment). This estimator must be greater than 0.7 to consider a domain homogeneous, but not too close to 1, which would mean that some questions are redundant.

Reproducibility will be studied by calculating ICC using analysis of variance for repeated measurement. An ICC of 0.65 is the minimum required for reproducibility. A Bland and Altman graph will be also done [35,36].

#### Secondary objectives

1. To longitudinally assess QoL of cancer patients until death using QLQ-C15 PAL, the French version of QUAL-E, and of Missoula Vitas Quality of Life Index (MVQOLI) Statistical analyses will be conducted to investigate longitudinal changes of each QoL dimension: description of missing QoL scores; missing QoL questionnaires and scores will be studied and classified according to Diggle and Rubins' method to determine whether the missing data were random or not: Missing Completely At Random, Missing At Random, Missing Not At Random [30,31,37,38].

Univariate and multivariate Logistic regression will be done to construct a clinical model to predict missing QoL and to investigate the random nature of missing QoL data. The numbers of missing scores will be described according to the patient subgroups and compared using ANOVA and Mixed Anova for repeated measurement.

### Longitudinal analyses

QoL will be described at each follow-up and then longitudinally analyzed using a mixed model analysis of variance for repeated measurements to investigate and test a time trend for QoL [39-41]:

These models will have to take into account non-random missing data.

With simple imputation methods for sensitivity analyses:

Mean QoL score imputation: Means will be calculated for the whole population and for specific patient profiles (cancer location, age, sex, and so on) at each follow-up and then will be imputed for patients with missing QoL scores

Extreme QoL score imputation. This method could be used when the missing data are due to a pejorative event such as death or disease progression.

With 'pattern mixture' models [42,43]:

Qualitative variables will be created to describe missing QoL score profiles and to define patterns for these analyses (no missing score, intermittent and drop-out profiles).

Time until definitive QoL score deterioration:

Analyses of time until a definitive QoL score deterioration (TUDD) will be estimated using the Kaplan-Meier method [44].

The TUDD of a score will be defined as the time interval between randomization and the first decrease in the MCID score ≥ 5 points compared to the QoL score at inclusion with no further improvement in QoL score ≥ 5 points or in the case of patient who dropped out after a ≥ 5-point decrease, resulting in missing data.

Patients will be censored at the last QoL follow-up when no ≥ 5-point deterioration in the QoL score from baseline will be observed or for patients with a ≥ 5-point deterioration, but with a subsequent ≥ 5-point improvement as compared to baseline QoL score.

Patients with no available QoL scores will be included in the TUDD analysis, but censored immediately.

2. To assess the impact of therapeutic strategies on QoL at the end of life Since treatments and care strategies will be longitudinally collected, for exploratory purposes only, we will compare longitudinal QoL according to these strategies using mixed model analysis of variance and Log Rank tests and Cox models for the time to definitive QoL deterioration analyses.

QoL will be described at each follow-up and compared at the inclusion (using Wilcoxon rank test) and then longitudinally analyzed using mixed model analyses of variance for repeated measurement [39-41] to test for the following: a therapeutic strategy effect whatever the follow-up; time effect whatever the therapeutic strategy; interactions between therapeutic strategy and follow-up; and effects of other clinical factors. The best model will be chosen according to the smaller Akaike's information criterion (AIC).

As for the TUDD approach, it will be compared with Log-Rank tests. Univariate and multivariate Cox analyses will be done to compute hazard ratios with 95% CI.

3. To assess the added value of spontaneous QoL completion to detect a clinically significant change in this setting

The proportion of patients who report at least one MCID during follow-up using the Jaeschke transition question will be compared according to the group allocated by randomization using univariate and multivariate logistic regressions.

We will also compute effect size (ES) and standardized response mean (SRM) to explore sensitivity to change according to spontaneous completion of a QoL questionnaire or not. We aim to demonstrate that with spontaneous completion of QoL questionnaires, ES and SRM will be improved.

For the sensitivity analysis, time to definitive QoL deterioration will also be estimated including this spontaneous completion of QoL questionnaires.

Analyses will be performed on an intent-to-treat principle.

4. To assess the effect of questionnaire order on QoL levels Longitudinal QoL analyses will be done as described above QoL will be described at each follow-up and compared at inclusion and then longitudinally analyzed using the mixed model analysis of variance for repeated measurements [39-41]. For the TUDD approach, it will be compared with Log-Rank tests. Univariate and multivariate Cox analyses will be done to compute hazard ratios with 95% CI.

Analyses will be performed on an intent-to-treat principle.

5. To define a period of 'end of life' QoL dimensions that strongly correlate with a patient's health status (physical dimensions, fatigue, pain) should decrease at the end of life, whereas dimensions (psychological, spirituality) more related to the end of life could remain stable and/or could slowly improve. These patterns in longitudinal QoL changes will be captured using mixed model analysis of variance for repeated measurement, and time until definitive QoL score deterioration.

### Data management

Data will be collected on CRF. The principal investigator in each center will be responsible for his or her data. Then data will be computerized and validated with Capture System software (Clinsight, Cenon, France) according to the specifications defined for this study.

## Discussion

The aim of this validation study will be to allow the assessment of QoL at the end of life in order to improve care for cancer patients. At this stage of the disease, patients require the support of clinicians and other caregivers for discussion, for psychological support and exchange in order to improve satisfaction and wellbeing. This support could help patients to cope with the end of life. Life completion and preparation for death are essential for patients' wellbeing and QoL at the end of life [45]. In this context, an external intervention could help to improve QoL dimensions, especially dimensions related to function, emotional status, anxiety, depression, and preparation for the end of life [46]. Moreover QoL questionnaires could be used as a communication tool to facilitate discussion about difficult subjects that the patient and/or the clinician would not have approached spontaneously [47]. Furthermore, QoL evaluations at the end of life and related discussions could improve the use of therapies and could contribute to appropriate care strategies [1,48].

In this context, the absence of validated tools to assess QoL in French cancer patients at the end of life must be resolved. This is one of the major preliminary steps to evaluate QoL in these patients. If our study fails to confirm the psychometric validity of these translated tools, we may have to investigate the possibility of creating a specific QoL tool for French cancer patients at the end of life.

In this case the creation of a new QoL questionnaire for French cancer patients at the end of life will be supported by the '*Qualité de vie et cancer*' clinical research platform. This new tool will be developed in agreement with reference methodology.

We think that these specific questionnaires could be useful for the evaluation of therapeutic approaches in future clinical trials or for everyday use in palliative care units. The ultimate goal should be to improve QoL of these patients who are trying to cope with end-of-life concerns. The perceptions, expectations, and experience of patients as well as physicians collected during this study could serve to improve the use of these QoL tools.

## Trial status

Between December 15, 2010 and April 5, 2011 30 patients have been included in five centers to check the understanding of the questions as well as their acceptability. Qualitative analyses of these pre-test data confirmed that questionnaires and translations were acceptable and that the study is feasible. Rewording of some items was the only adjustment needed with respect to understanding of the translation.

At the time of submission of the manuscript, the longitudinal prospective study to cross-culturally adapt the English questionnaires QUAL-E and Missoula Vital Quality of Life Index (MVQOLI), and to validate their psychometric properties in the end-of-life setting is ongoing. Nine centers were open and active and 12 patients out of the goal of 372 have been included since November 8, 2011.

## Abbreviations

EoL: End of life; QoL: Quality of life; ICF: Informed Consent form; CRF: Case report form; CRA: Clinical research assistant; IC: Intraclass correlation coefficients; MCID: Minimal clinically important difference; PPS: Performance palliative scale; ANOVA: Analysis of variance; TUDD: Time until a definitive QoL score deterioration.

## Competing interests

The authors declare that they have no competing interests.

## Authors' contributions

Conception and design: A-LP, FK, MM, and FB; manuscript writing: A-LP and FB; manuscript reviewing: FK, MM, VB, JMC, and Bd'A. All authors read and approved the final manuscript.
